# 6-Methyl­pyridin-3-amine

**DOI:** 10.1107/S1600536808040531

**Published:** 2008-12-13

**Authors:** Kai Zhu, Ning Xun, Ping Wei, Ping-Fang Han

**Affiliations:** a College of Life Science and Pharmaceutical Engineering, Nanjing University of Technology, Xinmofan Road No. 5 Nanjing, Nanjing 210009, People’s Republic of China

## Abstract

In the mol­ecule of the title compound, C_6_H_8_N_2_, the methyl C and amine N atoms are 0.021 (2) and 0.058 (2) Å from the pyridine ring plane. In the crystal structure, inter­molecular N—H⋯N hydrogen bonds link the mol­ecules.

## Related literature

For a related structure, see: Sawanishi *et al.* (1987 ▶). For bond-length data, see: Allen *et al.* (1987 ▶).
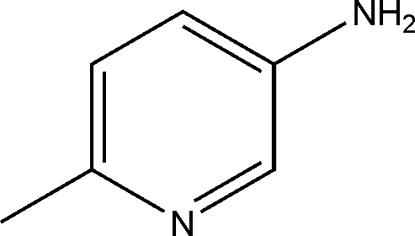


## Experimental

### 

#### Crystal data

C_6_H_8_N_2_
*M* *_r_* = 108.14
Monoclinic, 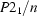

*a* = 8.4240 (17) Å
*b* = 7.0560 (14) Å
*c* = 10.658 (2) Å
β = 105.23 (3)°
*V* = 611.3 (2) Å^3^
*Z* = 4
Mo *K*α radiation
μ = 0.07 mm^−1^
*T* = 294 (2) K
0.30 × 0.20 × 0.10 mm

#### Data collection

Enraf–Nonius CAD-4 diffractometer
Absorption correction: ψ scan (North *et al.*, 1968 ▶) *T*_min_ = 0.978, *T*_max_ = 0.993
1183 measured reflections
1106 independent reflections
746 reflections with *I* > 2σ(*I*)
*R*_int_ = 0.059
3 standard reflections frequency: 120 min intensity decay: 1%

#### Refinement

*R*[*F* ^2^ > 2σ(*F* ^2^)] = 0.057
*wR*(*F* ^2^) = 0.154
*S* = 1.02
1106 reflections
73 parameters
H-atom parameters constrained
Δρ_max_ = 0.19 e Å^−3^
Δρ_min_ = −0.19 e Å^−3^

### 

Data collection: *CAD-4 Software* (Enraf–Nonius, 1989 ▶); cell refinement: *CAD-4 Software*; data reduction: *XCAD4* (Harms & Wocadlo, 1995 ▶); program(s) used to solve structure: *SHELXS97* (Sheldrick, 2008 ▶); program(s) used to refine structure: *SHELXL97* (Sheldrick, 2008 ▶); molecular graphics: *ORTEP-3 for Windows* (Farrugia, 1997 ▶); software used to prepare material for publication: *WinGX* (Farrugia, 1999 ▶).

## Supplementary Material

Crystal structure: contains datablocks global, I. DOI: 10.1107/S1600536808040531/hk2583sup1.cif

Structure factors: contains datablocks I. DOI: 10.1107/S1600536808040531/hk2583Isup2.hkl

Additional supplementary materials:  crystallographic information; 3D view; checkCIF report

## Figures and Tables

**Table 1:** Hydrogen-bond geometry (Å, °)

*D*—H⋯*A*	*D*—H	H⋯*A*	*D*⋯*A*	*D*—H⋯*A*
N2—H2*B*⋯N1^i^	0.86	2.29	3.131 (3)	165

Symmetry code: (i) 
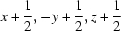
.

**Fig. 1.**
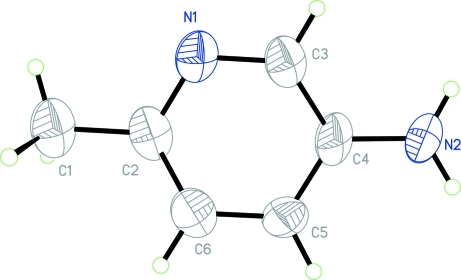
The molecular structure of the title molecule, with the atom-numbering scheme. Displacement ellipsoids are drawn at the 30% probability level.

**Fig. 2.**
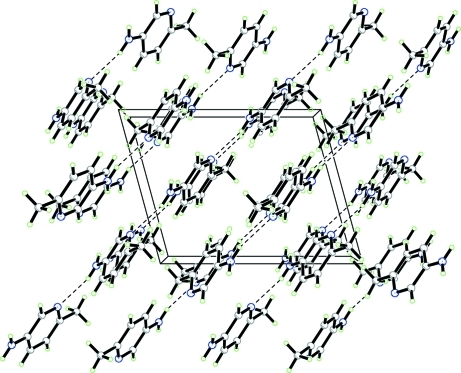
A partial packing diagram of the title compound. Hydrogen bonds are shown as dashed lines.

## supplementary crystallographic information

### Comment

Some derivatives of 3-pyridinecarboxylic acid are important chemical materials. We report herein the crystal structure of the title compound.

In the molecule of the title compound (Fig. 1) the bond lengths (Allen *et al.*,  1987) and angles are within normal ranges. Atoms C1 and N2 are 0.021 (2) Å and 0.058 (2) Å away from the pyridine ring plane.

In the crystal structure, intermolecular N-H···N hydrogen bonds (Table 1) link the molecules, in which they may be effective in the stabilization of the structure.

### Experimental

For the preparation of the title compound, bromine (17.3 g) was added slowly to sodium hydroxide solution (303 ml, 5%), and then 3-pyridinecarboxamide (13 g) was added in about 20 min at 273-278 K. The mixture was heated in an oil bath at 343-353 K for 4 h. The product was extracted with CH_2_Cl_2_, washed with water and dried (yield; 8 g, 77.6%) (Sawanishi *et al.*,  1987). Crystals suitable for X-ray analysis were obtained by slow evaporation of a methanol solution.

### Refinement

H atoms were positioned geometrically, with N-H = 0.86 Å (for NH_2_) and C-H = 0.93 and 0.96 Å for aromatic and methyl H, respectively, and constrained to ride on their parent atoms with U_iso_(H) = xU_eq_(C,N), where x = 1.5 for methyl H, and x = 1.2 for all other H atoms.

### Crystal data

**Table:**

C_6_H_8_N_2_	*F*(000) = 232
*M**_r_* = 108.14	*D*_x_ = 1.175 Mg m^−^^3^
Monoclinic, *P*2_1_/*n*	Mo *K*α radiation, λ = 0.71073 Å
Hall symbol: -P 2yn	Cell parameters from 25 reflections
*a* = 8.4240 (17) Å	θ = 10–12°
*b* = 7.0560 (14) Å	µ = 0.07 mm^−^^1^
*c* = 10.658 (2) Å	*T* = 294 K
β = 105.23 (3)°	Block, colorless
*V* = 611.3 (2) Å^3^	0.30 × 0.20 × 0.10 mm
*Z* = 4	

### Data collection

**Table:**

Enraf–Nonius CAD-4 diffractometer	746 reflections with *I* > 2σ(*I*)
Radiation source: fine-focus sealed tube	*R*_int_ = 0.059
graphite	θ_max_ = 25.3°, θ_min_ = 2.8°
ω/2θ scans	*h* = 0→9
Absorption correction: ψ scan (North *et al.*, 1968)	*k* = 0→8
*T*_min_ = 0.978, *T*_max_ = 0.993	*l* = −12→12
1183 measured reflections	3 standard reflections every 120 min
1106 independent reflections	intensity decay: 1%

### Refinement

**Table:**

Refinement on *F*^2^	Secondary atom site location: difference Fourier map
Least-squares matrix: full	Hydrogen site location: inferred from neighbouring sites
*R*[*F*^2^ > 2σ(*F*^2^)] = 0.057	H-atom parameters constrained
*wR*(*F*^2^) = 0.154	*w* = 1/[σ^2^(*F*_o_^2^) + (0.05*P*)^2^ + 0.5*P*] where *P* = (*F*_o_^2^ + 2*F*_c_^2^)/3
*S* = 1.02	(Δ/σ)_max_ < 0.001
1106 reflections	Δρ_max_ = 0.19 e Å^−^^3^
73 parameters	Δρ_min_ = −0.19 e Å^−^^3^
Primary atom site location: structure-invariant direct methods	

### Special details

**Table:**

Geometry. All e.s.d.'s (except the e.s.d. in the dihedral angle between two l.s. planes) are estimated using the full covariance matrix. The cell e.s.d.'s are taken into account individually in the estimation of e.s.d.'s in distances, angles and torsion angles; correlations between e.s.d.'s in cell parameters are only used when they are defined by crystal symmetry. An approximate (isotropic) treatment of cell e.s.d.'s is used for estimating e.s.d.'s involving l.s. planes.
Refinement. Refinement of *F*^2^ against ALL reflections. The weighted *R*-factor *wR* and goodness of fit *S* are based on *F*^2^, conventional *R*-factors *R* are based on *F*, with *F* set to zero for negative *F*^2^. The threshold expression of *F*^2^ > σ(*F*^2^) is used only for calculating *R*-factors(gt) *etc*. and is not relevant to the choice of reflections for refinement. *R*-factors based on *F*^2^ are statistically about twice as large as those based on *F*, and *R*- factors based on ALL data will be even larger.

### Fractional atomic coordinates and isotropic or equivalent isotropic displacement parameters (Å^2^)

**Table:**

	*x*	*y*	*z*	*U*_iso_*/*U*_eq_	
N1	0.8161 (3)	0.0759 (3)	0.11504 (18)	0.0480 (6)	
N2	1.0351 (3)	0.4188 (3)	0.3558 (2)	0.0595 (7)	
H2A	0.9692	0.5137	0.3361	0.071*	
H2B	1.1237	0.4285	0.4177	0.071*	
C1	0.8752 (4)	−0.2454 (4)	0.0569 (3)	0.0616 (8)	
H1B	0.7734	−0.2235	−0.0076	0.092*	
H1C	0.8626	−0.3507	0.1105	0.092*	
H1D	0.9602	−0.2730	0.0148	0.092*	
C2	0.9206 (4)	−0.0734 (4)	0.1390 (2)	0.0483 (7)	
C3	0.8578 (3)	0.2310 (4)	0.1886 (2)	0.0474 (7)	
H3A	0.7857	0.3333	0.1709	0.057*	
C4	0.9987 (3)	0.2519 (4)	0.2884 (2)	0.0478 (7)	
C5	1.1033 (3)	0.0962 (4)	0.3130 (2)	0.0503 (7)	
H5A	1.2009	0.1007	0.3788	0.060*	
C6	1.0608 (4)	−0.0656 (4)	0.2385 (2)	0.0544 (7)	
H6A	1.1290	−0.1713	0.2566	0.065*	

### Atomic displacement parameters (Å^2^)

**Table:**

	*U*^11^	*U*^22^	*U*^33^	*U*^12^	*U*^13^	*U*^23^
N1	0.0679 (13)	0.0494 (13)	0.0253 (10)	−0.0054 (11)	0.0098 (9)	0.0006 (10)
N2	0.0703 (15)	0.0579 (15)	0.0413 (13)	0.0013 (12)	−0.0013 (11)	−0.0182 (11)
C1	0.096 (2)	0.0527 (17)	0.0366 (14)	−0.0081 (15)	0.0191 (14)	−0.0067 (13)
C2	0.0835 (18)	0.0426 (14)	0.0242 (12)	−0.0001 (13)	0.0234 (12)	0.0044 (11)
C3	0.0707 (16)	0.0446 (14)	0.0302 (13)	0.0036 (12)	0.0190 (12)	0.0017 (11)
C4	0.0772 (17)	0.0489 (15)	0.0206 (11)	−0.0019 (13)	0.0187 (11)	−0.0033 (11)
C5	0.0622 (15)	0.0554 (16)	0.0301 (12)	0.0096 (13)	0.0062 (11)	0.0047 (12)
C6	0.0844 (19)	0.0481 (15)	0.0326 (13)	0.0087 (14)	0.0190 (13)	0.0038 (12)

### Geometric parameters (Å, °)

**Table:**

N1—C3	1.338 (3)	C1—H1D	0.9600
N1—C2	1.353 (3)	C2—C6	1.365 (4)
N2—C4	1.371 (3)	C3—C4	1.378 (4)
N2—H2A	0.8600	C3—H3A	0.9300
N2—H2B	0.8600	C4—C5	1.390 (4)
C1—C2	1.487 (3)	C5—C6	1.383 (4)
C1—H1B	0.9600	C5—H5A	0.9300
C1—H1C	0.9600	C6—H6A	0.9300
			
C3—N1—C2	117.9 (2)	N1—C3—C4	125.4 (2)
C4—N2—H2A	120.0	N1—C3—H3A	117.3
C4—N2—H2B	120.0	C4—C3—H3A	117.3
H2A—N2—H2B	120.0	N2—C4—C3	121.6 (2)
C2—C1—H1B	109.5	N2—C4—C5	122.5 (2)
C2—C1—H1C	109.5	C3—C4—C5	115.9 (2)
H1B—C1—H1C	109.5	C6—C5—C4	119.2 (2)
C2—C1—H1D	109.5	C6—C5—H5A	120.4
H1B—C1—H1D	109.5	C4—C5—H5A	120.4
H1C—C1—H1D	109.5	C2—C6—C5	121.3 (3)
N1—C2—C6	120.2 (2)	C2—C6—H6A	119.3
N1—C2—C1	118.1 (2)	C5—C6—H6A	119.3
C6—C2—C1	121.7 (3)		
			
C3—N1—C2—C6	2.2 (3)	N2—C4—C5—C6	−178.1 (2)
C3—N1—C2—C1	−179.6 (2)	C3—C4—C5—C6	−0.3 (4)
C2—N1—C3—C4	−0.5 (4)	N1—C2—C6—C5	−2.9 (4)
N1—C3—C4—N2	177.4 (2)	C1—C2—C6—C5	178.9 (2)
N1—C3—C4—C5	−0.4 (4)	C4—C5—C6—C2	1.9 (4)

### Hydrogen-bond geometry (Å, °)

**Table:**

*D*—H···*A*	*D*—H	H···*A*	*D*···*A*	*D*—H···*A*
N2—H2B···N1^i^	0.86	2.29	3.131 (3)	165

Symmetry codes: (i) *x*+1/2, −*y*+1/2, *z*+1/2.
